# Atlanta Academy of Medicine

**Published:** 1878-03

**Authors:** J. G. Westmoreland

**Affiliations:** Reporter


### Reports of Societies.

### ATLANTA ACADEMY OF MEDICINE.

J. G. WESTMORELAND, M.D., REPORTER.

ATLANTA, GA., February 18, 1878.

Dr. J. F. Alexander, President, in the chair.

Dr. L. G. Alexander reported a case of hypertrophic elongation of the cervix uteri. The case had been treated, previous to his taking charge of it, by a pessary. The neck, which is one and a half to two inches long, owing to the irritation caused by the pessary, was found engorged and tender, requiring treatment to allay it. Amputation, he thinks the only reliable remedy for elongation, and prefers the galvano-cautery for the purpose.

Dr. J. F. Alexander inquired of members as to the cause of leucorrhœal discharges so frequently met with in female children.

Dr. Baird thought that such discharge frequently occurred in young girls from the irritation of ascarades, which find their way into the vagina from the rectum.

Dr. Wilson thought leucorrhœa often occurs in young females about the time of first appearance of the catamenia, and is probably the result of the unusual engorgement of the organs of generation, which necessarily comes on at this period.

Dr. Baird alluded, in a few remarks, to the existence of ascarades in the rectum of children, which he meets with frequently. The cases which he has been called on to treat have been relieved readily by the ordinary anthelmintic preparation, calomel and santonin, used for the lumbricoides.

Dr. Wilson asked the experience of members with santonin in regard to its poisonous effects. He had used one-grain doses in a child of eight years old, and observed cerebral symptoms, which he attributed to the remedy.

Dr. Baird thought the dose small for the age mentioned, and that the unpleasant symptoms were probably the result of intestinal irritation.

Dr. Simpson agreed with Dr. Baird in attributing the symptoms mentioned by Dr. Wilson to some enteric trouble, and not to the poisonous effects of santonin.

Dr. L. G. Alexander had seen chilous urine in children, to whom santonin had been given, and which he supposed had been produced by the remedy. He had also seen children whose vision had been perverted by the use of santonin, so as to give a bluish color to objects.

